# Aeromonas australiensis sp. nov., isolated from irrigation water

**DOI:** 10.1099/ijs.0.055368-0

**Published:** 2013-08

**Authors:** Max Aravena-Román, Roxana Beaz-Hidalgo, Timothy J. J. Inglis, Thomas V. Riley, Antonio J. Martínez-Murcia, Barbara J. Chang, Maria Jose Figueras

In the abstract of the above paper (doi:10.1099/ijs.0.040162-0), the type strain LMG 26707^T^ is erroneously cited as LMG 2670^T^.

doi:10.1099/ijs.0.055368-0

